# Effects of *Moringa oleifera* leaves on hemoglobin and serum retinol levels and underweight status among adolescent girls in rural Bangladesh

**DOI:** 10.3389/fnut.2022.959890

**Published:** 2022-07-22

**Authors:** Mansura Khanam, Kazi Istiaque Sanin, Gulshan Ara, Razia Sultana Rita, Anika Bushra Boitchi, Fahmida Dil Farzana, Md. Ahshanul Haque, Tahmeed Ahmed

**Affiliations:** ^1^Icddr, b, Nutrition and Clinical Services Division, Dhaka, Bangladesh; ^2^Department of Global Health, University of Washington, Seattle, WA, United States; ^3^Department of Public Health Nutrition, James P. Grant School of Public Health, Bangladesh Rehabilitation Assistance Committee University, Dhaka, Bangladesh

**Keywords:** Moringa, hemoglobin, vitamin A, underweight, adolescent girls

## Abstract

**Objectives:**

*Moringa oleifera* has been used for centuries due to its medicinal properties and health benefits. The plant has antifungal, anti-viral, and anti-inflammatory properties. We aimed to evaluate the effect of consumption of Moringa leaves, along with a regular diet on serum hemoglobin and retinol and underweight status among rural Bangladeshi adolescent girls.

**Methods:**

This school-based quasi-experimental study involved 226 adolescent girls (12–14 years-old). Intervention group (*n* = 113) received a meal comprising rice, concentrated dal, and fried potato with Moringa pakora (oil-fried snack); the control group (at a different school in an adjacent area with similar population demographics) received calorie-matched meal without Moringa pakora for 6 months. We used generalized liner regression (GLM) analysis, to explore the effect of the intervention among the groups between baseline and endline.

**Results:**

Mean age of the intervention and control groups were 12.7 ± 0.7 and 13.3 ± 0.8 years, respectively. After adjusting for maternal education, absenteeism, asset index, BMI-for-age Z-score, GLM regression showed significant positive changes in hemoglobin (intervention vs. control: coef = 0.41, *P* = 0.010) and serum retinol (coef = 0.27, *P* = 0.00). No significant changes in weight was observed between groups.

**Conclusion:**

Consumption of Moringa leaves has the potential to improving hemoglobin and serum retinol level and should be encouraged as regular diet.

## Introduction

Adolescence, a unique period of human growth linking childhood and adulthood, includes 10–19 year-olds and is further divided into early adolescence (10–14-years-old) and late adolescence (15–19-years-old) (1). This period is considered the second window of opportunity to mitigate or even reverse the ill effects of inadequate development and nutritional deficiencies that may have existed since early childhood (2). Adolescents have additional nutritional requirements compared to adults, as they gain about 40% of their adult weight and 15% of their adult height during this period (3). Moreover, adolescent girls have a greater need for nutrient-dense food, to fulfill the demands caused by the onset of menstruation (4). Hence, insufficient nutritional intake during such crucial period has grave consequences throughout the reproductive years and beyond. Unfortunately, this larger demand for nutritious food almost always remains unmet among vulnerable adolescent girls residing in low-and-middle income countries (5).

Bangladeshi adolescents aged 10–19-years-old account for over one-fifth of the country's total population, including 14.4 million girls (6, 7). Two common factors that underlie the poor nutritional status of Bangladeshi adolescent girls are poor diets and early childbearing. Nearly 1 in 3 adolescent girls are thin, and 11% of girls are moderately or severely thin. The majority of them are micronutrient deficient, including iodine, zinc, and iron (8). A World bank report stated the proportion of adolescent girls having inadequate dietary diversity increased to 64% between 2012 and 2014 (9). About 80% of the kilocalories consumed per day by adolescents are derived from micronutrient-poor foods (70% from rice alone), particularly among girls from poor families (10). To mitigate this crisis, that report highlighted six prioritized interventions targeted at adolescent boys and girls that are proven to reduce undernutrition, which included weekly iron-folic acid (IFA) supplementation, school-based nutrition education, and provision of mid-day meals or fortified snacks at schools (11).

*Moringa oleifera* leaves have been found to contain the majority of essential nutrients required to maintain good health (12–15). The powdered form of the leaf is rich in multiple minerals and vitamins including iron, vitamin A (carotenoid), and vitamin C. Moreover, Moringa may help to resolve multiple malnutrition problems as it contains all essential amino acids, the building blocks for the proteins crucial to cell growth and metabolism (12, 16, 17). Based on Moringa's nutritional value and availability, and the emphasis given to the provision of fortified meals at school, we aimed to assess the effect of *M. oleifera* leaves to improve hemoglobin and retinol levels and reduce underweight status among adolescent girls in rural Bangladesh.

## Materials and methods

### Study design

We implemented a school-based intervention using a quasi-experimental design to evaluate the effect of consumption of Moringa leaves (along with regular diet and nutrition education) on the micronutrient status of adolescent girls in rural Bangladesh. To implement the study in a school-based setting, random assignment of students was not possible as it would be unethical to include some children from one class and not include other children.

### Study site

The study was conducted in Muktarpur union (the smallest rural administrative and local government units in Bangladesh) of Kaliganj sub-district, a semi-urban area located near to Dhaka, the capital of Bangladesh.

### Randomization procedure

We listed the schools in the region, consulted with the headmasters and school committee members, visited the facilities, and obtained the student's list. Of the six high schools in Muktarpur Union, three were excluded as only limited numbers of adolescent girls attended there. We discussed the purpose and objectives of the study with the local stakeholders and chairman. After getting their approval, we randomly selected one school for the intervention and another school as a control group.

### Study participants

**Figure 1:**
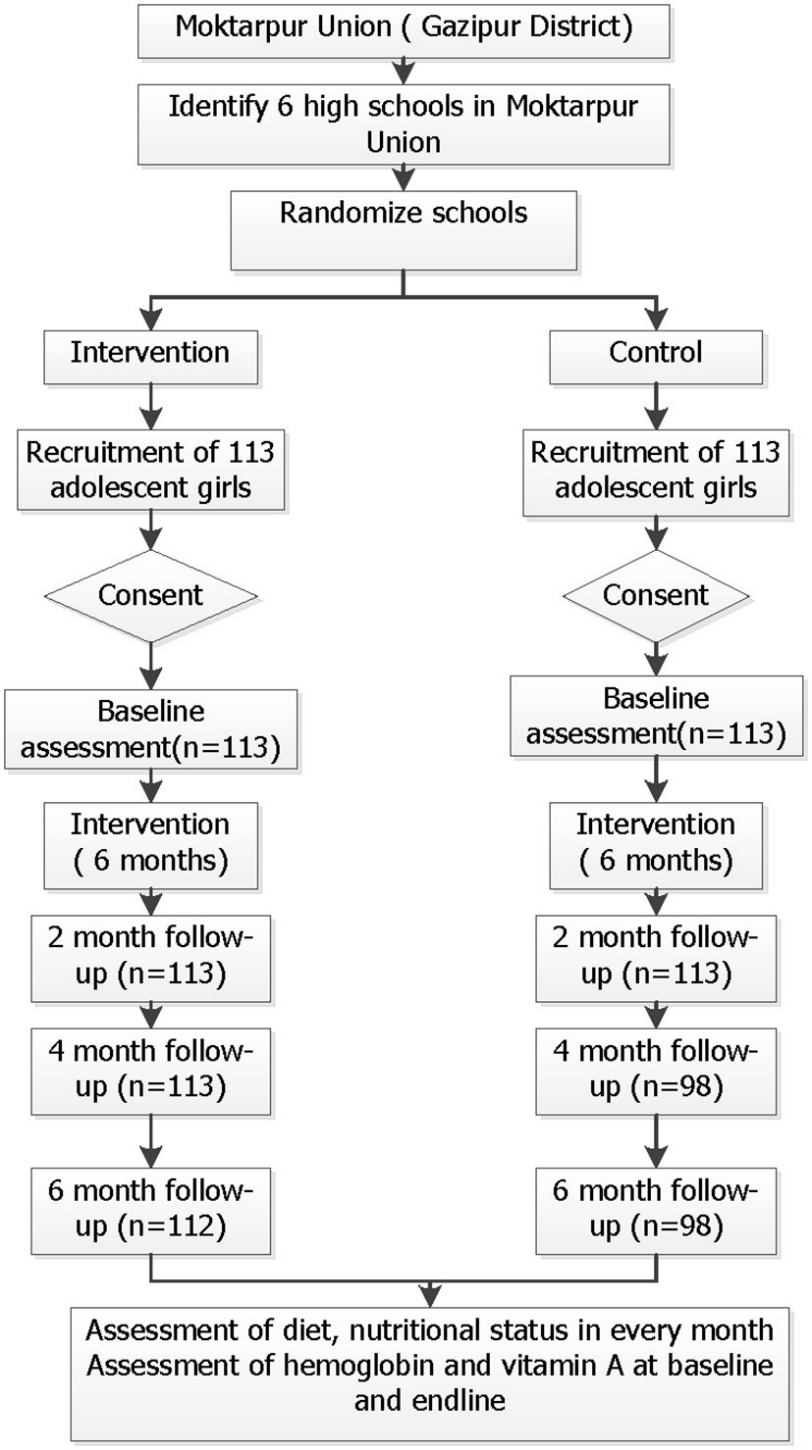
Study profile.

Our intervention was focused on the early adolescent period, when the rate of human growth is the highest outside early infancy. In Bangladesh, children in our target age group of 12–14 years usually study at class VI-VIII. After the selection of participating schools, list of female students in these classes were obtained from the schools. Age of the students was verified through birth certificate/immunization cards (if available) or admission information from the school archives. All female students in classes VI-VIII were screened. A total of 180 girls from intervention schools and 156 girls from control schools was screened and 226 adolescent girls (113 in each schools) completed the trial and returned follow-up questionnaires (Figure 1). Before starting the intervention, we conducted meetings with the parents at both schools to inform them of the nature of the intervention and data collection procedures. We visited the houses of all selected students to obtain the parents' consent and the student's assent. Only adolescent girls who gave assent themselves with their parents' consent were enrolled in the study.

#### Inclusion and exclusion criteria

Unmarried adolescent girls aged 12–14-years-old who attended the selected high schools were eligible for the study. Participants were excluded if they were taking any other nutritional supplements (vitamins and minerals), as this may affect the levels of hemoglobin and other micronutrients that we intended to measure. Adolescent girls with documented medical records of chronic disease were also excluded. However, we provided the meal to every girl due to ethical reason but collected data from only the recruited participants.

### Sample size

We assumed a mean hemoglobin level of 12.0 g/dL in the control group (18), mean hemoglobin level of 13.8 g/dL in the intervention group after the intervention, 80% power, 95% confidence interval, standard deviation (SD) of 4.50, and a 15% dropout rate. Based on the assumptions, the minimum sample size required was 226 individuals (113 in each arm). Furthermore, this sample size had 98% power to detect a change in serum retinol (control: 1.51; intervention: 1.74; SD: 0.42) and 87% power to detect a change in mean weight (control: 45.10; intervention: 48.10; SD: 7.0) between arms.

### Intervention

The intervention package included (1) a school-based mid-morning meal containing Moringa leaves (150 g) cooked traditionally as pakora (oil-fried snack), and (2) behavior change communication (BCC) on nutrition and dietary habits, personal health, and hygiene issues that magnify the risk of malnutrition for 6 months.

Each adolescent girl from the intervention group received Moringa leaves cooked as pakora along with 30 g rice and 25 g concentrated dal, once daily, for 5 days/week at tiffin time. The control group received 30 g rice, 25 g concentrated dal and 25 g fried potato (*aluvaji*), once daily for 5 days/week. Each tiffin box was numbered so that leftover food could be measured daily for each participant. A standard food record form was used to measure food compliance. The tiffin meals provided to both arms supplied 411 kilocalories.

#### Preparation of the meals

We recruited two local cooks who were responsible for preparing the meals daily under direct supervision of the field staff. Two field staff monitored and measured the cooking procedure and placed the meals in the food boxes using measuring cups to measure 30 g of rice and 25 g of concentrated dhal. To prepare Moringa pakora, 150 g granules of fresh *sajna* (Moringa) leaves were mixed with mashed lentils, and then onion, chili and salt were added and mixed to make a paste. A portion of ~50 g paste was fried as pakora. Each student received 3 pakora/ day. Three pakoras provided 282 kilocalories/person/day.

#### Behavior change communication (BCC)

Two locally recruited health workers conducted bi-monthly counseling on nutrition and dietary habits, personal health, and menstrual hygiene, targeting the female adolescents from both arms in small groups after the school day had ended. The duration of each session was usually 30–40 min.

### Data collection

A team of field staff was responsible for implementing the study under the guidance of the Investigator(s). Before starting the field activities, 5 days of intensive training was provided to the field staff. The emphasis of the training was interview techniques and understanding the questionnaire. Field staff also received training on anthropometric measurements following the standard WHO guidelines (19).

Before the survey, the questionnaire was thoroughly discussed, extensive field-testing was conducted, and necessary modifications were made. The structured questionnaire was used to collect information on socioeconomic status and demographics, health and morbidity, food consumption patterns, and other relevant information.

#### Household demographics

Information on ethnicity, religion, level of education of household head, occupation of household head, number of family members, ownership of the house, number of dwelling rooms, household construction materials, toilet facilities, sources of drinking water, household assets, land ownership, and household monthly expenditure were collected as key indicators of socioeconomic status.

#### Feeding history of adolescent girls

Data on food items were collected using an adapted version of FANTA's Minimum Dietary Diversity for Women. The categories are: (1) grains, white roots, tubers, and plantains; (2) pulses (beans, peas, and lentils); (3) nuts and seeds; (4) dairy; (5) meat, poultry, and fish; (6) eggs; (7) oils and fats; (8) fruits; (9) vegetables; and (10) condiments and seasonings, etc. (20).

#### Weighed food record

Consumption of the provided mid-morning meals was measured daily using a standard food record form to measure compliance to the food.

#### Anthropometric measurements

Trained field staff was collected anthropometric measurements (weight and height) monthly using established methods (21) and record these measurements on the questionnaire. The weight of each adolescent in school uniform without shoes were measured in kilograms using a portable Tanita Scale with an accuracy of 100 g. Height was recorded with the adolescents' head level with a horizontal Frankfurt plane (below the imaginary line from the lower border of the eye orbit to the auditory meatus) without shoes using a wooden height measuring board with a sliding head bar to the nearest 0.1 cm. All measurements had taken twice, unless the difference between the two readings is beyond acceptable accuracy. All instruments were calibrated every morning against a standard weight and a height stick.

#### Morbidity questionnaire

Trained field staff asked all participants standard questions about recent morbidity within the previous 15 days using pre-coded questionnaires designed for recording specific morbidity symptoms. The morbidity recall period was 2 weeks. Urinary tract infections and symptoms of menstrual problems and experiences regarding school absenteeism was also collected monthly.

#### Assessment of hemoglobin

Anemia assessment was done by considering the hemoglobin concentration of capillary blood using HemoCue 301 device (HemoCue AB, Angleholm, Sweden). Trained research staff was conducted the assessments. After asepsis by chlorhexidine gluconate at 0.5%, the disposable lancet on the fingertip (middle or ring or index) made a puncture. The first drop of blood was discarded. The second drop made to form by gentle pressure. Once the sample is formed, the micro cuvette was dipped into it to fill it up with the blood sample by capillary action.

#### Assessment of serum retinol

Peripheral blood samples were collected from all participants in the intervention and control groups at baseline, 3 months and endline. The blood samples were labeled using bar-coded identification labels specifically created for this study that correspond to the subject's identification number. Thus, the laboratory had easily identified which particular cluster scan been tested in a batch and thus minimize repeated freeze/thaw cycles. A sample record/handover form, including the name of the participant, ID number, sample ID number and type of analysis, was completed. Samples were transported to the nutritional biochemistry laboratory in Dhaka in a temperature-controlled cool box and stored in a −70°C freezer at the laboratory until blood parameters were quantified by high-performance liquid chromatography (HPLC).

### Delivery of the intervention

Trained staff were responsible for delivering the food in the classrooms with the help of two locally recruited field staff. They went to the classroom during tiffin time, provided the meals to the students, and informed the students not to throw food out as the leftover food was measured. Each tiffin box had an identifier number for each participant to measure the remaining food. Every class also had male students, and we also offered them the food, if they wanted it. However, their intake was not measured or monitored. Total intervention period was 6 months.

### Ethical approval

This study (PR-19060) was approved by the Research Review Committee and Ethical Review Committee, the two compulsory components of the institutional review board of the International Center for Diarrhoeal Disease Research, Bangladesh (icddr,b). Written informed consent was obtained from all study participants (>18-years-old) and/or assent (<18-years-old) from their parents/guardians prior to enrolment.

### Study outcomes

The primary outcomes were the differences in the mean changes in the adolescent girls' hemoglobin and retinol levels from baseline to endline between the intervention group and control group. The secondary outcome was the difference in weight gain between the intervention group and the control group.

### Data management

A senior research officer was responsible for regular observations at the schools and checking the data for validity and completeness. The staff independently repeated the data collection for 3–5% of the study participants on the same day using a field-tested format.

After completion of data collection, data were entered into Microsoft (MS) Access software. Maximum validation rules were set in the data system to prevent errors during data entry. After completing the entry, data were transferred to Stata (Release 14, College Station, Texas 77845, USA: StataCorp LP) software.

### Data analysis

#### Descriptive analysis

Statistical plots such as histograms, scatter plots were used for data visualization. Descriptive statistics were used to compare frequencies and proportions for categorical variables, and means and standard deviations for symmetric quantitative variables.

#### Exploratory analysis

We used the Student's *t*-test and the chi-square test to compare means and explore the associations among categorical variables. Coefficients were generated by generalized linear regression modeling (GLM), in which a dependent variable is regressed on the set of hemoglobin and retinol levels. Effect of intervention was adjusted for variables those were associated with the outcome during univariate regression such as maternal age, sex of household head, maternal education, BMI for age, wealth index and absentism of school. *P*-values < 0.05 were considered significant for all tests.

## Results

Table 1 compares the household characteristics of the participating girls in the intervention and control groups. More than 90% of the household heads were male, and their main religion was Islam. The households in both groups had a similar number of rooms that were used for sleeping. More parents of participants from the control group had completed primary education, whereas more parents in the intervention group had completed secondary education. A higher proportion of mothers worked in the intervention group (12.39%) than in the control group (9.73%). Around 35.4% of the participants' fathers were unemployed in both groups; farming and business were the main occupations of the majority of the working fathers. About 73.4 and 72.6% of households in the intervention and control group used personal tube wells as the main source of drinking water. A higher percentage of households in the control group used sanitary latrines than in the intervention group.

**Table 1:** Household characteristics of the participants by group.

**Household characteristics**	**Intervention** **[*****N*** = **113]**	**Control** **[*****N*** = **113]**
**Sex of household head**		
Male	79.7 (90)	85.8 (97)
Female	20.3 (23)	14.2 (16)
**Religion**		
Islam	111 (98.2)	108 (95.6)
Hinduism	2 (1.8)	5 (4.4)
**Number of rooms used for sleeping**		
1–2	65 (57.5)	66 (58.4)
>2	48 (42.5)	47 (41.6)
**Mother's occupation**		
Service holder	14 (12.4)	11 (9.7)
Housewife	99 (87.6)	102 (90.3)
**Father's occupation**		
Agricultural laborer	21 (18.5)	27 (23.9)
Service	14 (12.4)	9 (7.9)
Business	14 (12.4)	21 (18.6)
Factory worker	9 (8.0)	7 (6.2)
Construction laborer	7 (6.2)	9 (8.0)
Unemployed	48 (42.5)	40 (35.4)
**Mother's education**		
Illiterate	15 (13.3)	8 (7.08)
Primary completed	49 (43.36)	70 (61.9)
Secondary and higher	49 (43.36)	35 (30.9)
**Father's education**		
Illiterate	27 (23.9)	8 (7.08)
Primary completed	42 (37.1)	81 (71.7)
Secondary and higher	44 (38.9)	24 (21.2)
**The main source of drinking water**		
Own tube well	83 (73.4)	82 (72.6)
Other's tube well	2 (1.8)	6 (5.3)
Community tube well	1 (0.9)	0 (0)
Supply water (piped)	0 (0)	1 (0.9)
Deep tube well	27 (23.9)	24 (21.2)
**Toilet facility of household members**		
Sanitary with flush	4 (3.6)	17 (15.1)
Sanitary without flush	47 (41.6)	58 (51.3)
Pucca/pit	57 (50.4)	37 (32.7)
Kutcha/Hanging	5 (4.4)	1 (0.9)
**Toilet facility used for children**		
Sanitary with flush	4 (3.6)	17 (15.1)
Sanitary without flush	47 (41.6)	58 (51.3)
Pucca/pit	55 (48.7)	34 (30.1)
Kutcha/Hanging	6 (5.2)	1 (0.9)
Others	1 (0.9)	3 (2.6)
**Share of toilet facility with other households**		
Yes	11 (9.7)	27 (23.9)
No	102 (90.3)	86 (76.1)
**Ownership of the house**		
Owns the house	102 (90.3)	110 (97.3)
Rented	2 (1.8)	1 (0.9)
In kind	9 (7.9)	2 (1.8)
Wealth quintile^*^		
Poor	20 (17.7)	26 (23.0)
Poorer	24 (21.2)	21 (18.6)
Middle	20 (17.7)	25 (22.1)
Richer	22 (19.5)	23 (20.4)
Richest	27 (23.9)	18 (15.9)
**Respondent characteristics**		
Age (Y) (mean ± SD)	12.7 ± 0.7	13.3 ± 0.8
Year of schooling (mean ± SD)	6.52 ± 0.50	7.43 ± 1.09
Weight (kg)	42.4 ± 9.7	42.6 ± 8.3
Height (cm)	148.8 ± 7.5	149.7 ± 5.7
Hemoglobin (g/dL)	12.0 ± 0.7	11.8 ± 0.9
Serum retinol (μmol/l)	1.3 ± 0.3	1.4 ± 0.9

* *Wealth quintile: The household wealth quintile was constructed using household asset data obtained from the Socioeconomic Status questionnaire. From these asset-related dichotomous variables, a common factor score for each household was generated using polychoric principal components analysis in STATA software. After ranking based on their score, we divided first principal component score into quintiles to create five categories where the first category represents the poorest household, and the fifth category represents the wealthiest household*.

The mean age of the adolescent girls in the intervention and control groups was 12.7 and 13.3 years, respectively; this difference was not statistically significant. The mean weight and height of the girls were similar in both groups. The mean hemoglobin level was 12.0 and 11.8 g/dL for the intervention and control groups, respectively. Serum retinol was similar in both groups (1.3 μmol/l).

**Figure 2:**
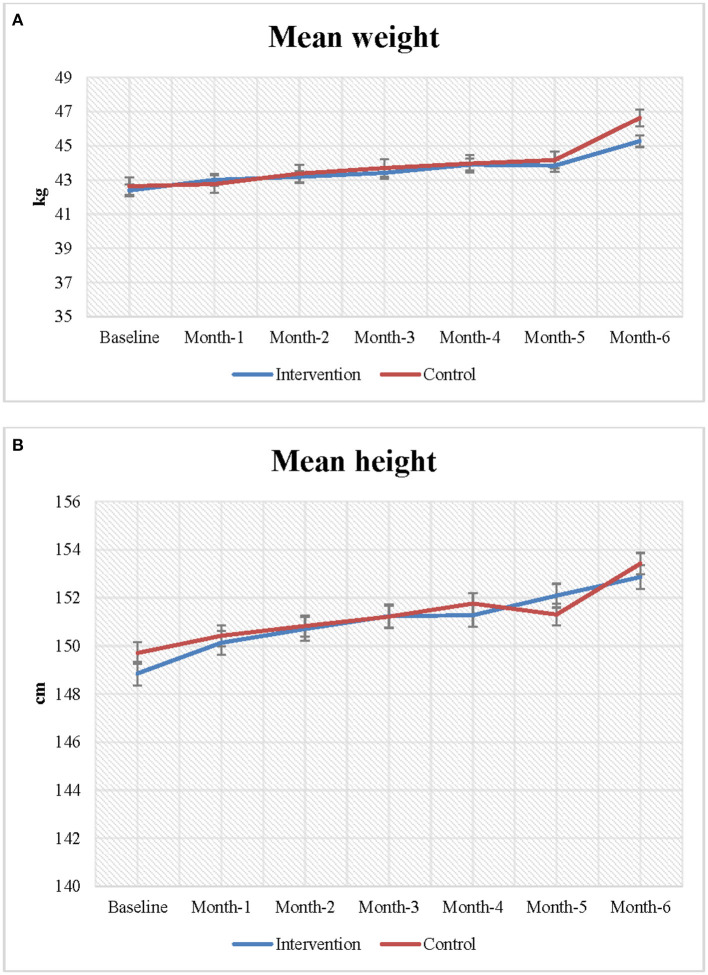
**(A,B)** Changes in nutritional status among the study participants.

Figures 2A,B presents the mean weight and height of the adolescent girls at baseline and endline. Increases in both height and weight were observed during follow-up in both the intervention and control groups; however, the changes in height and weight between baseline and endline were greater in the intervention group than the control group. The mean weight of the adolescent girls was 42.0 kg at baseline. At endline, the mean weight of the intervention group increased more than that of the control group (46.64 vs. 45.28 kg).

At baseline, the girls in the intervention group (148.85 cm) were marginally shorter than the girls in control group (149.70 cm). However, at the end of the follow-up, the height of the girls in the intervention group had increased more than the height of the girls in the control group (153.43 vs. 152.87 cm). On average, participants in the intervention group became 0.56 cm taller than the girls in the control group, however, the association was not statistically significant.

The primary outcomes were mean hemoglobin and serum retinol levels, as presented in Table 2. We performed generalized linear regression modeling to assess the effect of the intervention by adjusting for (i) differences between the control and intervention groups (potential confounding factors) and (ii) temporal trends in the outcome unrelated to the intervention. After adjusting for maternal education, household head sex, absenteeism, asset index, and BMI-for-age *Z*-score, GLM analysis showed a significant positive change in the hemoglobin level (intervention compared to control: coef = 0.41; 95% CI: 0.14, 0.76; *P* = 0.010). A significant positive change in the retinol level was also observed between the intervention group compared to the control group (coef = 0.27; 95% CI: 0.14, 0.36; *P* = 0.00). We did not observe any significant impact of the intervention on weight gain among the study participants.

**Table 2:** Changes in biomarkers between the intervention and control groups.

	**Baseline**			**Endline**				
**Indicators**	**Intervention (*****n*** = **113)**	**Control** **(*****n*** = **113)**	**Diff**	**Intervention (*****n*** = **112)**	**Control (*****n*** = **99)**	**Diff^a^**	**Coef^b^**	***P*** **-value**
Haemoglobin^b^ (g/dL)	12.04	11.78	0.31	13.31	12.59	0.72	0.41 (0.14, 0.76)	0.009
Retinol (μmol/l)^b^	1.32	1.40	−0.07	1.38	1.19	0.19	0.27 (0.14, 0.36)	0.000
Weight (kg)^c^	42.39	42.64	0.25	46.64	45.28	−1.36	1.41 (−1.91, 4.72)	0.406
BMI for age^c^	−0.09	−0.24	−0.16	0.20	−0.05	−0.25	0.09 (−0.34, 0.51)	0.674

a *Difference between control and Intervention at baseline and endline*.

b *Effects of the intervention over 6 months of follow-up using GLM with adjustment for maternal age, sex of household head, maternal education, BMI for age, wealth index, and absentism of school*.

c *Effects of the intervention over 6 months of follow-up using GLM with adjustment for maternal age, sex of household head, maternal education, wealth index, and absentism of school*.

## Discussion

This study aimed to assess the effect of consumption of Moringa leaves (alongside normal staple foods) on the nutritional status of adolescent girls in Bangladesh. To our best knowledge, this is the first study to assess the nutritional effects of Moringa in Bangladesh, which is widely available in the country and traditionally used to treat fever and diarrhea. However, there is limited scientific evidence to confirm the benefits of this potential plant-based food. Our school-based, quasi-experimental study demonstrates the beneficial effects of regular consumption of Moringa leaves on Bangladeshi female adolescents. Consumption of Moringa leaves as part of a daily nutritious snack, combined with nutrition BCC, significantly improved the average hemoglobin and retinol levels of the female adolescents aged 12–14 years.

The measurement of average hemoglobin level from our study echoes with the result of school-going children from the last National Micronutrient Survey done in 2011–12. At baseline, average hemoglobin level was higher in the intervention group compared to the control group, though, the differences were not statistically significant. In our study, although both groups had an increment of the hemoglobin level at endline, the intervention group experienced a significant improvement. These results are in agreement with previous studies that showed consumption of Moringa leaves increased the mean hemoglobin level from 11.43 to 12.36 g/dL (22) and 10.43 g/dL to 10.85 gm/dl among women of reproductive age (15–45-years-old) from a poor segment of the Indian population (23). Moringa leaves have a high vitamin C content, which increases the bioavailability of iron (24). Vitamin C has also been shown to improve the absorption of iron from non-haem sources by up to 4-fold (25). As vitamin C and iron combine to form ferrous-ascorbate complexes that are soluble and easily absorbed, fresh vegetables and fruits high in vitamin C are particularly effective in improving hemoglobin level. Several studies showed that different side dishes made from Moringa also increase the absorption of non-haem iron from foods consumed; for instance, Suzana et al. found that side dishes and fruits and vegetables affected the haematocrit values among school aged-children that consumed Moringa in Ghana (26). Based on our positive finding; fresh Moringa leaves could potentially be promoted as a natural food-based supplement for improving hemoglobin level among women of reproductive age.

Our study demonstrates regular consumption of Moringa leaves significantly improved the serum retinol of school-going adolescent girls. Serum retinol is required for effective utilization of iron and to maintain normal hemoglobin levels (27). Significant correlations have been explored between the serum retinol concentration and hemoglobin, indicating a possible relationship between vitamin A status and the use of iron for haematopoiesis (28). *Moringa* leaves have a total carotene content of 40,000 μg and beta carotene content of 19,000 μg/100g, thus having equivalent bioavailability as synthetic vitamin A (29, 30). At the baseline, serum retinol was higher in our control group. However, with intervention, the serum retinol level significantly increased in the intervention group. A study conducted among 35 young Mexican children aged 17–35 months old demonstrated that consumption of moringa leaves improved the storage of retinol in the body (31). Another study conducted in Ghana found that supplementation of a cereal-legume blended flour with Moringa leaves increased mean vitamin A levels among children aged 4–12-years-old; however, this population was deficient in vitamin A at baseline (32). Glover-Amengo et al. (32) showed that the vitamin A status of school-age children could be improved by supplementing their diets with Moringa leaf powder. About 53.3% of school-age-children in Bangladesh have mild vitamin A deficiency (0.35–0.70 μmol/l). Our findings suggest that regular consumption of moringa leaves may be effective for the vitamin A deficient population in Bangladesh or in other vitamin A-deficient populations. As locally available food material, Moringa leaves may represent a cost-effective strategy to combat the prevalent problem of nutritional deficiencies and underweight status among adolescent girls in Bangladesh. Incorporating recipes with Moringa leaves in the daily diet could be employed as a preventive and maintenance strategy for better health outcomes.

A major strength of this study was the high response rate (98%) in each group, which enabled a fair statistical comparison. The Hemocue devices used to measure hemoglobin level were calibrated every day; the retinol samples were transported to the nutritional biochemistry laboratory at the icddr,b and analyzed in a blinded manner. Nevertheless, our work has some limitations. A limitation of the study was its quasi-experimental design, as it was not possible to mask the fieldworkers responsible for delivering the meals to the participants and recoding the data, or the participants to their group allocation.

## Conclusion

Our school-based intervention showed that addition of Moringa leaves to the diet significantly improved the hemoglobin and retinol levels, among adolescent girls in Bangladesh. Incorporating recipes with Moringa leaves in the daily diet may provide a simple preventive and maintenance strategy to ameliorate micronutrient deficiency during this critical period of female growth and development, in achieving better health outcomes.

## Data availability statement

The raw data supporting the conclusions of this article will be made available by the authors, without undue reservation.

## Ethics statement

This study (PR-19060) was approved by the Research Review Committee and Ethical Review Committee, the two compulsory components of the Institutional Review Board of the International Center for Diarrhoeal Disease Research, Bangladesh (icddr,b). Written informed consent was obtained from all study participants (>18-years-old) and/or assent (<18-years-old) from their parents/guardians prior to enrolment. Written informed consent to participate in this study was provided by the participants' legal guardian/next of kin.

## Author contributions

MK and KIS conceptualized the study, drafted the original manuscript, and revised the final version of the manuscript. KIS contributed to drafting the manuscript and result interpretation. MK, KIS, GA, RSR, ABB, FDF, MAH, and TA had contributed to data analyses, interpretation, and write-up of the manuscript. All authors contributed to the article and approved the submitted version.

## Funding

This research study was funded by icddr,b's core donors and Swedish International development Cooperation Agency (Sida), Sweden, grant number GR:01455.

## Conflict of interest

The authors declare that the research was conducted in the absence of any commercial or financial relationships that could be construed as a potential conflict of interest.

## Publisher's note

All claims expressed in this article are solely those of the authors and do not necessarily represent those of their affiliated organizations, or those of the publisher, the editors and the reviewers. Any product that may be evaluated in this article, or claim that may be made by its manufacturer, is not guaranteed or endorsed by the publisher.
